# Myricetin as an Antivirulence Compound Interfering with a Morphological Transformation into Coccoid Forms and Potentiating Activity of Antibiotics against *Helicobacter pylori*

**DOI:** 10.3390/ijms22052695

**Published:** 2021-03-07

**Authors:** Paweł Krzyżek, Paweł Migdał, Emil Paluch, Magdalena Karwańska, Alina Wieliczko, Grażyna Gościniak

**Affiliations:** 1 Department of Microbiology, Faculty of Medicine, Wroclaw Medical University, 50-368 Wroclaw, Poland; emil.paluch@umed.wroc.pl (E.P.); grazyna.gosciniak@umed.wroc.pl (G.G.); 2 Department of Environment, Hygiene and Animal Welfare, Wroclaw University of Environmental and Life Sciences, 51-630 Wroclaw, Poland; pawel.migdal@upwr.edu.pl; 3 Department of Epizootiology and Veterinary Administration with Clinic of Infectious Diseases, Faculty of Veterinary Medicine, Wroclaw University of Environmental and Life Science, 50-366 Wroclaw, Poland; magdalena.karwanska@upwr.edu.pl (M.K.); alina.wieliczko@upwr.edu.pl (A.W.)

**Keywords:** *Helicobacter pylori*, coccoid forms, morphological transformation, myricetin, checkerboard assay, synergism, biofilm

## Abstract

*Helicobacter pylori*, a gastric pathogen associated with a broad range of stomach diseases, has a high tendency to become resistant to antibiotics. One of the most important factors related to therapeutic failures is its ability to change from a spiral to a coccoid form. Therefore, the main aim of our original article was to determine the influence of myricetin, a natural compound with an antivirulence action, on the morphological transformation of *H. pylori* and check the potential of myricetin to increase the activity of antibiotics against this pathogen. We observed that sub-minimal inhibitory concentrations (sub-MICs) of this compound have the ability to slow down the process of transformation into coccoid forms and reduce biofilm formation of this bacterium. Using checkerboard assays, we noticed that the exposure of *H. pylori* to sub-MICs of myricetin enabled a 4–16-fold reduction in MICs of all classically used antibiotics (amoxicillin, clarithromycin, tetracycline, metronidazole, and levofloxacin). Additionally, RT-qPCR studies of genes related to the *H. pylori* morphogenesis showed a decrease in their expression during exposure to myricetin. This inhibitory effect was more strongly seen for genes involved in the muropeptide monomers shortening (*csd3*, *csd6*, *csd4*, and *amiA*), suggesting their significant participation in the spiral-to-coccoid transition. To our knowledge, this is the first research showing the ability of any compound to synergistically interact with all five antibiotics against *H. pylori* and the first one showing the capacity of a natural substance to interfere with the morphological transition of *H. pylori* from spiral to coccoid forms.

## 1. Introduction

The use of antibiotics in human treatment is considered to be one of the greatest medical achievements of the 20th century [1]. Nevertheless, about 100 years after their widespread introduction, the applicability of antibiotics has decreased drastically. This phenomenon seems to be strongly influenced by the dynamic spreading of antibiotic-resistant microorganisms, especially multidrug-resistant and pandrug-resistant strains. Currently, the annual mortality of patients caused by antibiotic-resistant microorganisms is estimated at about 700,000, while statistical analyses indicate that in 2050 this number may rise to a dramatic 10 million deaths per year [2]. For this reason, the World Health Organization is trying to highlight the seriousness of the above situation and encourage scientists for the intensification of research on new therapies against antibiotic-resistant pathogens [3].

Resistance to antibiotics is a multifactorial phenomenon with a tendency of quick spreading in society [4]. Thus, fighting this requires a variety of strategies, including not only improving the management of currently available antibiotics, but also the application of alternative methods targeting pathogens. Among them, there is increasing interest in antivirulence therapies, i.e., the use of substances that interfere with the factor(s) being crucial for the pathogenicity of a given microorganism [4,5]. The compounds that hinder the production of pathogens’ virulence factors may deprive them of their ability to develop infections or defend themselves against the host, making them easier to eradicate.

**Figure 1:**
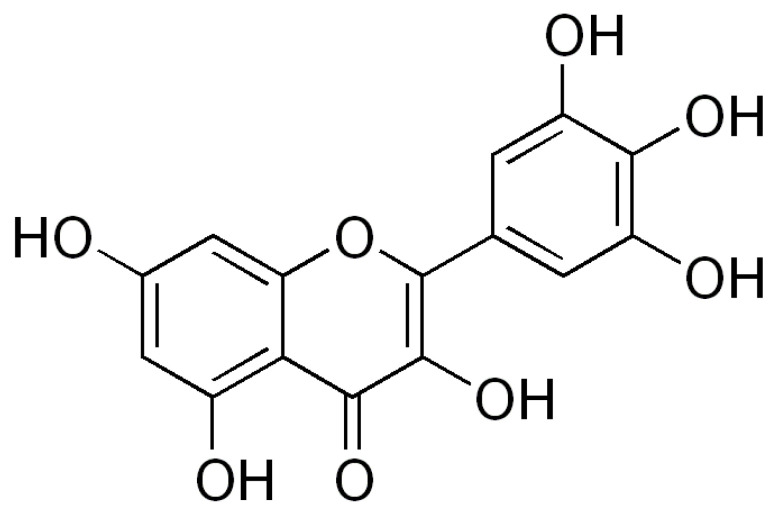
The chemical structure of myricetin (MYR; 3,5,7,3′,4′,5′-hexahydroxyflavone).

Within the natural substances with promising antivirulence properties, special attention is now paid to polyphenols, secondary plant metabolites characterized by multiples of phenol units [6,7]. This diverse group of compounds includes myricetin (MYR; 3,5,7,3′,4′,5′-hexahydroxyflavone, Figure 1), where, in recent years, a particularly high ability to inhibit a number of virulence factors has been demonstrated [8]. MYR was originally isolated from the bark of the *Myrica* tree, which gave the compound its name [9,10]. In its pure form MYR appears as yellow crystals, a property with which its initial application as a biopigment was related to [10]. Over the years, the usefulness of this compound in the fight against allergies, inflammations, hypertension, and diabetes has been recognized [8]. Additionally, a very promising antibacterial activity has been observed [8,9,10]. However, the antibacterial features of MYR are not related to the disturbance of microbial growth, but are rather associated with a capacity to decrease pathogenicity of microbes, including their cytotoxic action (production of lytic enzymes or toxins), eukaryotic cell colonization (adhesion and invasion), and defense mechanisms (e.g., biofilm formation) [11,12,13,14,15]. Therefore, MYR appears to be an ideal candidate for use in antivirulence therapies for difficult-to-treat pathogens.

*Helicobacter pylori* is a Gram-negative bacterium that colonizes the gastric mucosa [16,17,18]. Infections most often occur during early childhood and, in the absence of the appropriate treatment, are life-long. The gastric environment is an unfavorable niche and for this reason *H. pylori* has developed various adaptations enabling the effective colonization of this organ. Strains that produce multiple virulence factors are believed to have a greater potential to persistently infect the stomach [19]. The classically mentioned virulence determinants include urease [20], vacuolating toxin (VacA) [21], cytotoxic oncoprotein (CagA) [22], and numerous adhesins [23]. However, more and more often attention is paid to the morphological transformation of this bacterium as one of the key properties conditioning the survival of *H. pylori* [24]. The capacity to change from the spiral/rod-shaped form to the coccoid form is associated with a number of physiological changes in cells of this bacterium, which are accompanied by an increased ability to avoid detection from the host’s immune system and a significant reduction in antibiotic sensitivity [24,25]. It has even been indicated that the presence of coccoid *H. pylori* forms is an independent risk factor related to patients’ therapeutic failures [26,27] and that coccoid forms of this bacterium are able to produce virulence factors, as well [28]. Therefore, it seems that finding a substance capable of interfering with the process of morphological transformation of *H. pylori* may turn out to be very useful for increasing the degree of eradication of this pathogen [24,25].

Based on the above deduction, the aim of this study was to determine the influence of MYR on the morphological transformation of *H. pylori* and to determine the potential of MYR to increase the activity of classically used antibiotics against this pathogen.

## 2. Results

### 2.1. Antibacterial Activity of MYR

**Figure 2:**
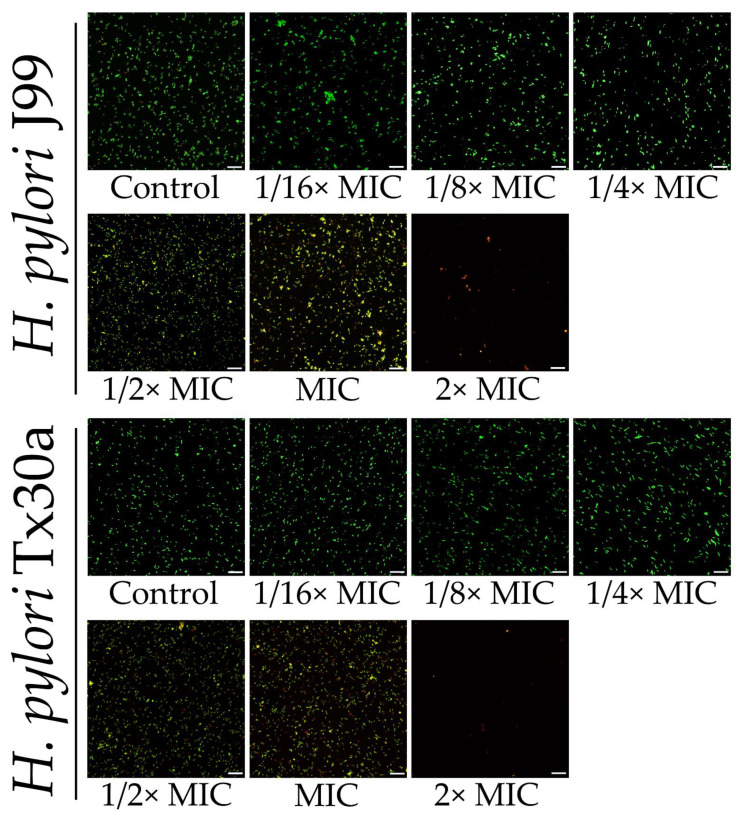
Representative fluorescence microscopy images of reference *H. pylori* J99 and Tx30a strains exposed to a gradient concentration of myricetin. The minimal inhibitory concentration (MIC) in both strains counts for 160 µg/mL, while minimal bactericidal concentration (MBC) is equal to 2× MIC and counts for 320 µg/mL. Bacterial cells were stained with a LIVE/DEAD kit. The scale bar shows 20 µm.

In the first stage of our research, we decided to determine the antibacterial activity of MYR against *H. pylori*. We observed that minimal inhibitory concentrations (MICs) and minimal bactericidal concentrations (MBCs) were convergent and counted for 160 and 320 µg/mL, respectively, for both tested strains (*H. pylori* J99 and Tx30a) (Table 1). As we were interested in establishing the antivirulence potential of MYR and not affecting the growth of *H. pylori*, we additionally conducted a fluorescence analysis of LIVE/DEAD-stained cells exposed to different concentrations of this compound. We observed that sub-minimal inhibitory concentrations (sub-MICs) of MYR up to 1/4× MIC had a marginal effect on the green/red fluorescence ratio of bacterial cells, but some antibacterial effect was noticeable starting from 1/2× MIC (Figure 2). Therefore, in further analyses we decided to focus on MYR in a range of 1/256×–1/4× MIC when checking its modulatory effect on the morphological transformation of *H. pylori*.

### 2.2. Inhibitory Effect of MYR against the Spiral-to-Coccoid Transformation

When assessing the ability of MYR to inhibit the transformation of *H. pylori* into coccoid forms, we applied serum starving conditions, a well-known factor stimulating this process [29,30]. The two reference *H. pylori* strains used in this study provide a good model to study this type of interaction. The highly virulent *H. pylori* J99 has a high potential for the spiral-to-coccoid transition, contrary to the *H. pylori* Tx30a, a non-pathogenic strain with the low transformative potential [31].

**Figure 3:**
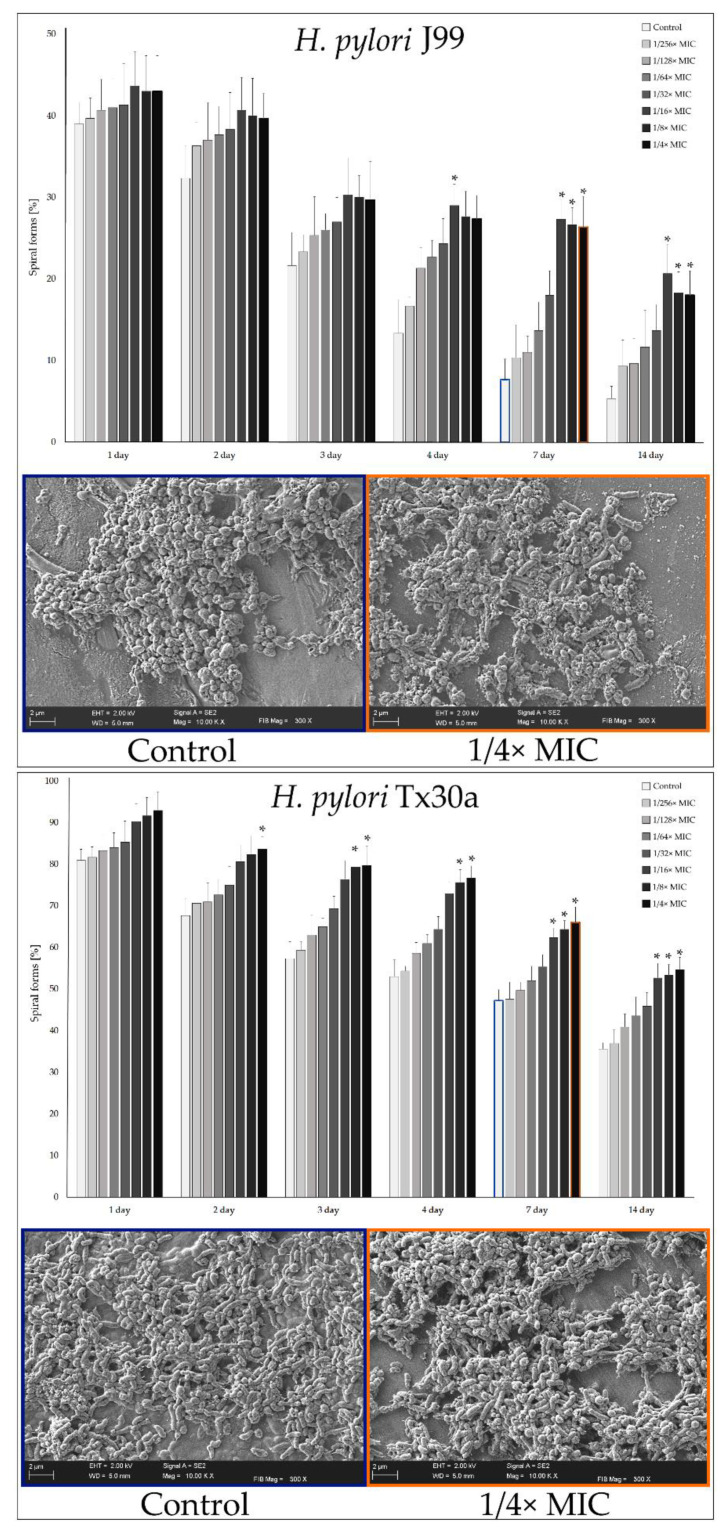
The concentration- and time-dependent activity of myricetin in inhibiting the transition of reference *H. pylori* J99 and Tx30 strains from spiral to coccoid forms during serum starving conditions. The MIC of myricetin for both strains is equal to 160 µg/mL. The initial number of spiral forms (a 0-h time point) was 90% and 98% for *H. pylori* J99 and Tx30a, respectively. Results are presented as means ± standard deviations from three independent biological experiments. * Indicates statistically significant difference (*p* ≤ 0.05).

We observed that the effect of slowing down the transformation of *H. pylori* is dependent on both the concentration and the duration of exposure to MYR. The *H. pylori* J99 tended to reduce the number of spiral forms very quickly in the conditions we used (a decrease from 90% to 39%–43% in the first day) (Figure 3). The 7- and 14-day exposure of this strain to 1/16×–1/4× MIC of MYR allowed us to maintain about 3–4-times higher amount of spiral forms than in the control samples. The *H. pylori* Tx30a, in line with previous assumptions, lowered the number of spiral forms much slower than *H. pylori* J99 (from 98% to 81%–93% in the first day) (Figure 3). Here, differences between control samples and these exposed to sub-MICs of MYR were statistically significant from the 2nd day of culture, while the difference was most noticeable on the 7th and 14th day of incubation (approx. 1.5-times higher number of spiral forms than in the control). Our observations, showing a significantly higher number of spiral forms in samples treated with 1/4× MIC of MYR for 1 week, were additionally confirmed by scanning electron microscopy (SEM) (Figure 3). In conclusion, we showed that MYR at concentrations between 1/16× MIC and 1/4× MIC has a strong potential for modulating the speed of transformation into coccoids by *H. pylori*.

### 2.3. Inhibitory Effect of MYR against Biofilm Formation

**Figure 4:**
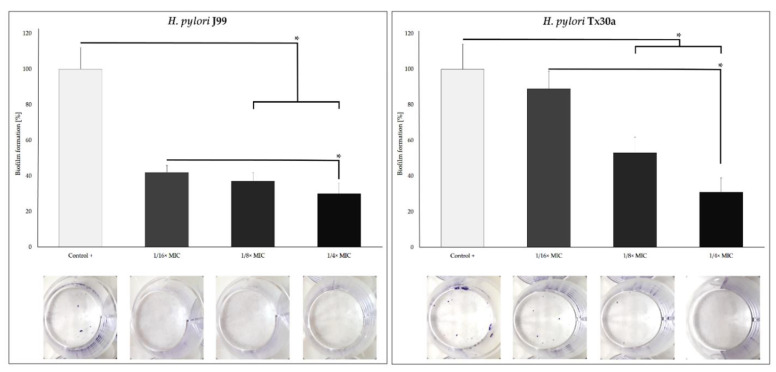
Anti-biofilm activity of myricetin against reference *H. pylori* J99 and Tx30a strains measured by a crystal violet staining method. The MIC of myricetin for both strains is equal to 160 µg/mL. Results are presented as means ± standard deviations from three independent biological experiments with three repetitions each. * Indicates statistically significant difference (*p* ≤ 0.05).

Keeping in mind that biofilm formation is another important feature that may facilitate antibiotic tolerance of *H. pylori* [32], we decided to use a crystal violet staining method to determine the ability of *H. pylori* to form this structure during the presence of 1/16×–1/4× MIC of MYR. We found that a 3-day incubation of this pathogen with sub-MICs of MYR caused the concentration-dependent inhibitory effect on biofilm development (Figure 4). Exposure to 1/8× and 1/4× MIC resulted in a 50% and 70% reduction in the biofilm formation of *H. pylori* Tx30a and 60% and 70% decrease for *H. pylori* J99, respectively.

### 2.4. Synergistic Activity of MYR with Antibiotics

In further stages of our research, we focused on the analysis of the potential of sub-MICs of MYR (1/16×–1/4× MIC, concentrations being the strongest spiral-to-coccoid inhibitors) to enhance the antibacterial activity of five clinically used antibiotics (amoxicillin, clarithromycin, tetracycline, metronidazole, and levofloxacin) against *H. pylori*.

**Figure 5:**
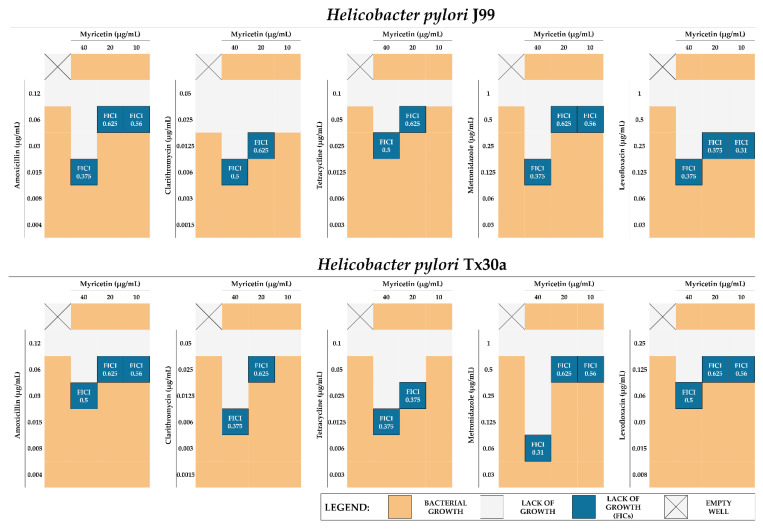
Graphical representation of the results of checkerboard assays testing synergistic interactions between myricetin and antibiotics against reference *H. pylori* J99 and Tx30a strains. The MIC of myricetin for both strains is equal to 160 µg/mL. The MICs for amoxicillin, tetracycline, and metronidazole for both strains count for 0.12, 0.1, and 1 µg/mL, respectively. The MICs for clarithromycin and levofloxacin are equal to 0.025 and 1 µg/mL for *H. pylori* J99, and 0.05 and 0.25 µg/mL for *H. pylori* Tx30a. The blue field marks the wells of the titration plates, in which no bacterial growth and a positive interaction between the tested compounds was observed. The interactions were interpreted based on the calculation of the fractional inhibitory concentration index (FICI), in which ≤0.5 and >0.5 but ≤1 was considered as synergism and additivity, respectively.

We discovered that 1/4× MIC of MYR is able to lower the MICs of all tested antibiotics by 4–16 times, which allows us to conclude its potential to synergistically enhance the antibiotics’ action (fractional inhibitory concentration indexes, FICI = 0.31–0.5) (Figure 5). The 1/8× MIC of MYR also increased the antimicrobial activity of all tested antibiotics, although in this case most of the interactions were additive (for most of them FICI = 0.625) (Figure 5). The 1/16× MIC of MYR additively increased the activity only of bactericidal antibiotics (amoxicillin, metronidazole, and levofloxacin; FICI = 0.56), but not bacteriostatic ones (clarithromycin and tetracycline, FICI = 2.0) (Figure 5). 

**Figure 6:**
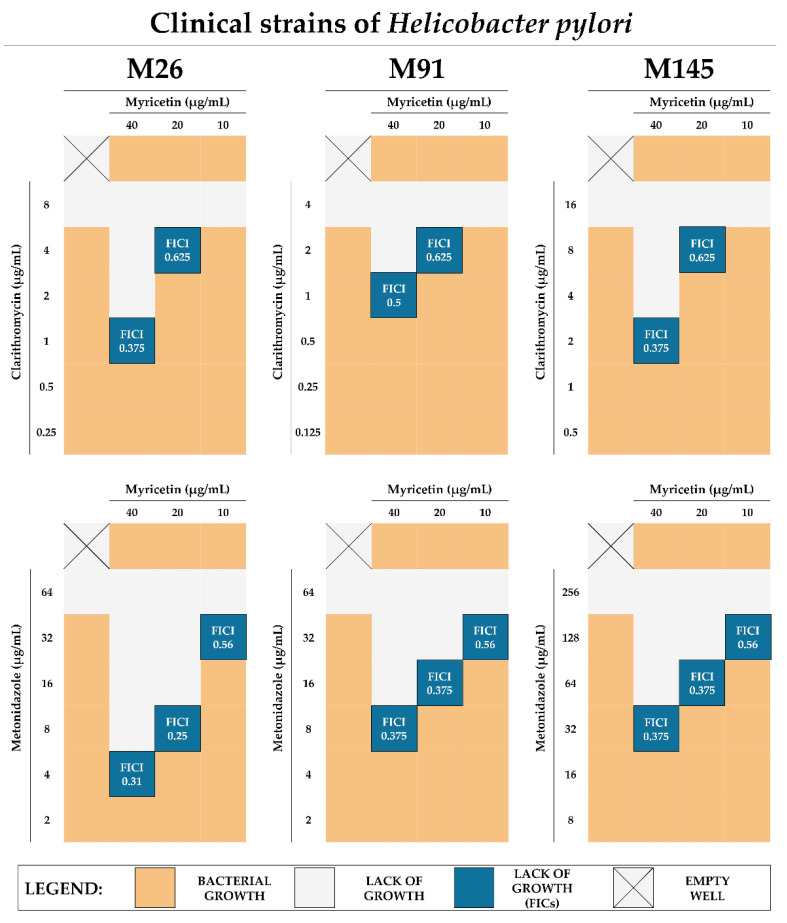
Graphical representation of the results of checkerboard assays testing synergistic interactions between myricetin and two selected antibiotics (clarithromycin and metronidazole) against clinical, double-resistant *H. pylori* strains (clarithromycin- and metronidazole-resistant). The MIC of myricetin for all strains is equal to 160 µg/mL. The MICs for clarithromycin count for 8, 4, and 16 µg/mL for *H. pylori* M26, M91, and M145, respectively. The MICs for metronidazole are equal to 64, 64, and 256 µg/mL for *H. pylori* M26, M91, and M145, respectively. The blue field marks the wells of the titration plates, in which no bacterial growth and a positive interaction between the tested compounds was observed. The interactions were interpreted based on the calculation of the FICI, in which ≤0.5 and >0.5 but ≤1 was considered as synergism and additivity, respectively.

Dual resistance to clarithromycin and metronidazole of *H. pylori* is considered to be the most important resistance profile determining therapeutic failures of this pathogen [33,34], therefore we extended our research with three clinical *H. pylori* strains with the above-mentioned resistance. Checkerboard assays determining the sub-MICs of MYR with clarithromycin or metronidazole confirmed our previously described observations, indicating synergism of 1/4× MIC of MYR with these antibiotics (FICI = 0.31–0.5) and suggesting that resistance to antibiotics of *H. pylori* is not adversely affecting the antibiotic-boosting effect of MYR (Figure 6).

**Figure 7:**
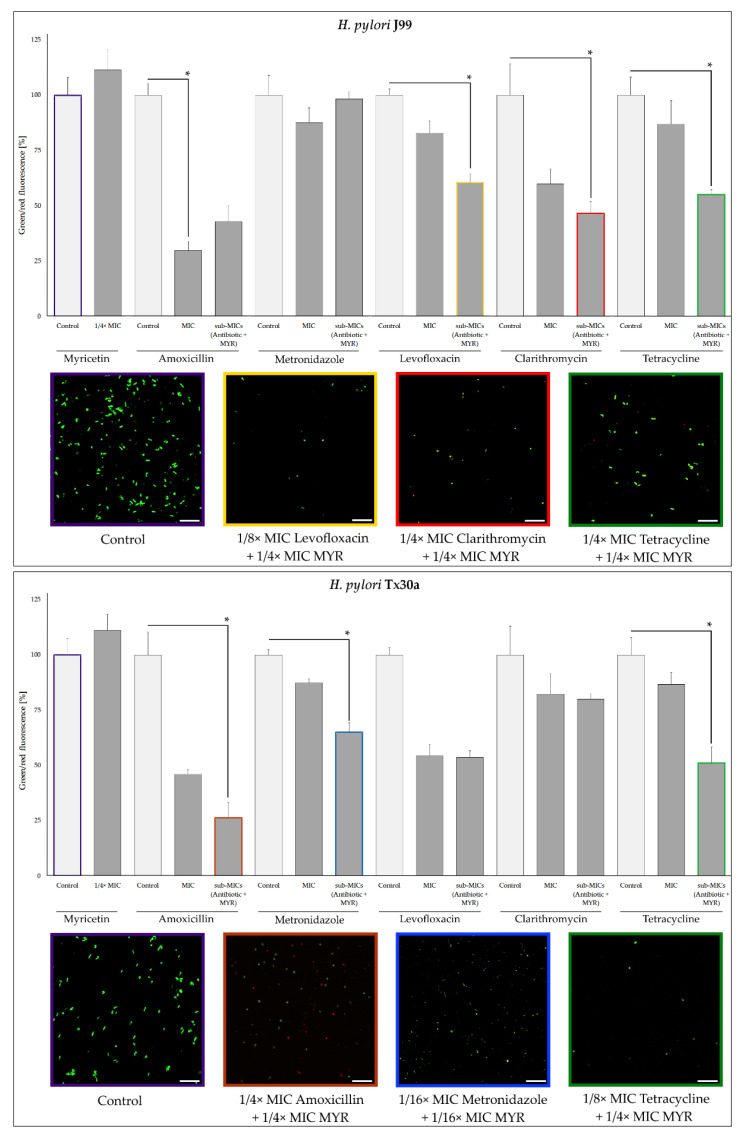
Graphical representation of changes in cell viability of reference *H. pylori* J99 and Tx30a strains when exposed to MICs of antibiotics or a combination of sub-minimal concentrations (sub-MICs) of the selected antibiotic with 1/4× MIC of myricetin (MYR). Fluorescence in the “control” and “1/4× MIC” samples collectively labeled as “myricetin” is the mean of all other experiments, being their joint element. Bacterial cells were stained with a LIVE/DEAD kit. The scale bar shows 20 µm. Results are presented as means ± standard deviations from three independent, biological experiments. * Indicates statistically significant difference (*p* ≤ 0.05).

To broaden the information on the synergism of MYR with antibiotics, we performed the fluorescence analysis of *H. pylori* J99 and Tx30a cells treated with MICs of antibiotics alone or in the combination with MYR (fractional inhibitory concentrations (FICs), being 1/4× MIC of MYR + sub-MICs of the selected antibiotic) (Figure 7). We observed that the green/red fluorescence ratio of bacterial cells was reduced in most samples compared to the controls. Among the 7/10 of biological samples in which we noticed a significant decrease in cell viability, six of them constituted samples of two combined substances (sub-MICs of both MYR + antibiotic) (Figure 7). These results show that MYR at 1/4× MIC is not only able to reduce the MIC values of all tested antibiotics while maintaining their antimicrobial activity, but even intensify this antibacterial activity against *H. pylori*.

### 2.5. Modulatory Effect of MYR on Genes Related to Morphogenesis

The last stage of our research was to determine the effect of a 3-day exposure of *H. pylori* on 1/4× MIC of MYR, the concentration that most effectively potentiated the action of antibiotics, on the expression of genes related to the morphogenesis of this pathogen. In this case, we selected genetical determinants involved in the peptidoglycan modeling, i.e., cleaving of cross-linking bridges of muropeptide dimers (*csd1, csd2*, and *csd3*) and shortening of muropeptide monomers (*csd3, csd6, csd4*, and *amiA*) [25]. In particular, the activity of genes associated with the second peptidoglycan rearrangement pathway seemed important to us as it has been suggested that the trimming of penta-, tetra-, and tripeptides to dipeptides is a molecular marker associated with the formation of spherical *H. pylori* forms [35,36,37].

**Figure 8:**
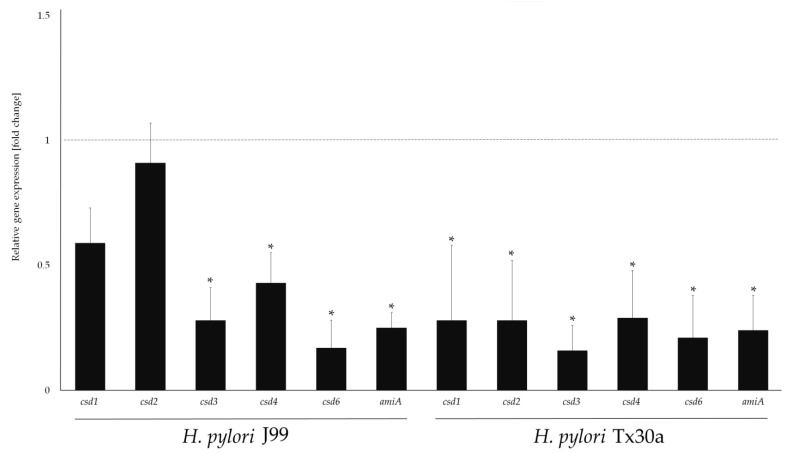
Modulatory activity of 1/4× MIC of myricetin (40 µg/mL) on the expression of the morphogenesis-associated genes of reference *H. pylori* J99 and Tx30a strains. Expression of the tested genes was related to the expression of the house-keeping gene—*ureA*, which presented sufficient stability during these studies (the difference between the expression in the control and experimental samples was less than 1). The dashed line indicates no change in gene expression. A 2-fold change in expression was considered statistically significant (*). Results are presented as means ± standard deviations from three independent, biological experiments.

The results of these analysis were similar for both *H. pylori* strains. Exposure to MYR caused a reduction in the expression of all tested genes with the inhibition most strongly observed for *csd3* (3.6- and 6.2-fold for *H. pylori* J99 and Tx30a, respectively), *csd6* (5.8- and 4.8-fold for *H. pylori* J99 and Tx30a, respectively), and *amiA* (about 4-fold for both *H. pylori* strains) (Figure 8). For *csd4*, we also observed some inhibitory effect (2.3- and 3.5-fold for *H. pylori* J99 and Tx30a, respectively), but not as strong as for the genes mentioned before (Figure 8). Despite the large convergence of the results obtained for both *H. pylori* strains, we observed some differences in the expression of *csd1* and *csd2*. Exposure of *H. pylori* J99 to 1/4× MIC of MYR had a marginal effect on the expression of these genes (less than a 2-fold change), while in *H. pylori* Tx30a this effect was significant (about a 3.5-fold reduction in their expression) (Figure 8).

## 3. Discussion

The main goal of pathogenic microorganisms is to achieve a preferred niche in the host, replicate and avoid destruction, and transfer to the next organism [38]. To obtain this, pathogens have developed the ability to produce many virulence determinants, some of which have been known and characterized widely for many years (toxins, lytic enzymes, siderophores, envelopes, etc.), while the importance of other, more sophisticated strategies has been recently described [38,39]. Bacterial morphology affects the capacity to survive stressful environmental conditions, colonize specific niches within hosts or escape the immune system detection [40,41]. Therefore, scientists speculate to consider the microbial shape and the possibility of its transformation as an important pathogenicity strategy. High morphological heterogeneity and the ability to change from spiral to coccoid forms is one of the key features of *H. pylori* associated with antibiotics tolerance and therapeutic failures [24,25]. For this reason, in this original paper, we attempted to inhibit this process in order to increase the sensitivity of *H. pylori* to classically used antibiotics.

In recent years, very promising antivirulence properties have been reported for MYR. Although this substance possesses some antibacterial action, its activity is described as relatively low. The MIC values of MYR reported by others are in the range of 62.5–256 µg/mL [13,42,43,44,45] and are in line with those obtained by us (MIC = 160 µg/mL) (Table 1). Moreover, it was observed that sub-MICs of this compound do not disturb the growth of microorganisms but may inhibit the production of many virulence factors, including sortase A of Gram-positive bacteria [46]; adhesins, proteases, and collagenases of *Porphyromonas gingivalis* [13]; suilysin of *Streptococcus suis* [14]; and invasiveness of *Salmonella* Typhimurium [15]. Research groups focusing on *Staphylococcus aureus* have also reported very promising results [11,12,42,47], demonstrating the capacity of MYR to disrupt adhesion, biofilm formation, and synthesis of hemolysin α and staphyloxanthin.

In this study, we were focused on determining the ability of MYR to interfere with the transformation of *H. pylori* into coccoid forms. We observed a concentration- and time-dependent slowing in the transition of *H. pylori* into spherical forms when treated with this compound (Figure 3). For example, an 1-week incubation under starving conditions resulted in 2–4 times (*H. pylori* J99) and 1.5 times (*H. pylori* Tx30a) higher amount of spiral forms during exposure to 1/16×–1/4× MIC of MYR than in control samples. These observations were additionally confirmed by scanning electron microscopy. Ideally, exposure of *H. pylori* to MYR would allow a complete and long-term inhibition of the spiral-to-coccoid transformation. Compared to spiral forms, coccoids have a lower level of metabolism, a tendency to self-aggregate and form biofilms, and a higher efflux pumps expression, all of which generate their antibiotic tolerance [24,32]. As demonstrated by Obonyo et al. (2012) [48] and Faghri et al. (2014) [49], preformed coccoid *H. pylori* forms require much higher concentrations of antimicrobial substances to be destroyed than spiral forms (even if these can transform to spherical forms over time). It seems that spiral forms accumulate sufficiently high concentrations of antimicrobial compounds in their interior that the subsequent conversion to coccoid forms no longer has a protective function and is rather an expression of cell death. Thus, on the basis of the above considerations and the deduction presented in a review by Krzyżek et al. (2020) [24], we conclude that the slowing down of the morphological transition might be sufficient to break down the coccoid-related antibiotic tolerance of *H. pylori*.

For MYR, apart from the affinity to diminish the virulence of various pathogens, the ability to enhance the activity of antimicrobial substances has also been demonstrated [43,44,47]. In our research, we showed very promising properties of MYR as an enhancer of antibiotics against *H. pylori*. We demonstrated the synergistic effect of 1/4× MIC of MYR in combination with all five tested antibiotics (amoxicillin, clarithromycin, tetracycline, metronidazole, and levofloxacin), allowing us to decrease 4–16 times the MIC values of these antibiotics (Figure 5 and Figure 6). In addition, observations made by fluorescence microscopy and LIVE/DEAD staining confirmed the results of the checkerboard assays (Figure 7). Based on our review published in 2020 [50], being a holistic revision of in vitro studies on *H. pylori* synergistic treatments, we can conclude that MYR is the first described compound capable of synergistically interacting with all five antibiotics against *H. pylori*. The enhancement of antibiotics’ activity was not related to the bacterial resistance profile as convergent results were obtained for both the two reference *H. pylori* strains (J99 and Tx30a) (Figure 5) and the three clinical, double-resistant *H. pylori* strains (M26, M91, and M145) (Figure 6). The targets of MYR in microbial cells are replication and transcription, which results from the inhibition of DNA and RNA polymerases [51,52,53]. It seems that the blockage of transcription may condition the decreased expression of genes related to the microbial stress response, making pathogens more sensitive to the action of antimicrobial substances [54]. For many representatives of polyphenols, the ability to interact and disrupt the function of bacterial cell membrane and/or cell wall has been demonstrated [55], and although this type of activity has not been demonstrated for MYR so far, we cannot conclusively state that this type of phenomenon did not occur in this case.

With RT-qPCR, we inspect the capacity of MYR to interfere with the expression of genes responsible for the *H. pylori* morphogenesis. Among the most important genetic determinants involved in *H. pylori* peptidoglycan rearrangement, there are two groups of genes related to this process [25]. The first group is responsible for cleaving cross-linking bridges of muropeptide dimers (*csd1, csd2*, and *csd3*) and the second is involved in muropeptide monomers shortening (*csd3, csd6, csd4*, and *amiA*). The *csd3* is associated with cutting pentapeptides into tetrapeptides, which are then used by *csd6* to produce tripeptides, which in turn are processed by *csd4* to create dipeptides [25]. The *amiA*, on the other hand, is associated with the coordination of functioning of these genes [36]. In the present study, we observed that MYR seems to have a stronger influence on the second stage of *H. pylori* murein modification (trimming of muropeptide monomers), with an approximately 4-fold reduction in the expression of these genes (Figure 8). This is consistent with the observations showing the importance of dipeptides accumulation in coccoid *H. pylori* forms [35,36,37], and the participation of *csd4* and *amiA* as determinants of this morphological transformation in both *H. pylori* [36,56] and *Campylobacter jejuni* [57]. Taking into account our results, we suspect that MYR may be responsible for the disturbance of the *csd3* and *csd6* activity (contributing to the decrease of substrates for the *csd4* functioning), as well as the inhibition of *amiA*, a gene coordinating the work of the aforementioned determinants. The interruption of morphogenesis may also affect other virulence traits, such as biofilm formation, as previously shown for *C. jejuni* [58]. Although the effect of MYR on the formation of *H. pylori* biofilm was not the main goal of our current original work, we undertook a preliminary verification on the influence of sub-MICs of MYR on the initial stages of this structure development. We observed that 1/4× MIC of MYR reduced the amount of *H. pylori* biofilm by 70% (Figure 4). These results encourage us to undertake further research on the modulating effects of MYR on *H. pylori* biofilms in the future.

An issue worth considering in the context of *H. pylori* therapy is the delivery method and the stability of substances in the gastric environment. MYR is a slightly acidic compound [59,60], which may positively influence its use in the therapy of *H. pylori*. In laboratory conditions simulating the stomach environment, it was shown that MYR had a very high level of stability with a t_1/2_ at pH = 2 of approx. 10 h [59]. The solubility was also enhanced by an acidic environment, with an increase in this parameter by 46- to 9-fold at pH between 1 and 3. Although MYR is found in many vegetables, fruits, nuts, and herbs [8], it is highly doubtful that these concentrations would be sufficient to achieve the desired therapeutic effect against *H. pylori*. However, it was shown that the use of carriers for MYR significantly improves its bioavailability [61,62,63,64]. One of the strategies may be the use of biocellulose, the utilization of which has been shown by our research group in ex vivo experiments in burning wounds/bones infections [65,66] and preliminary in vitro tests on *H. pylori* [67]. The applicability of this type of delivery system in *H. pylori* therapy with MYR will be determined by us in the future.

## 4. Materials and Methods

### 4.1. H. pylori Strains

Two reference *H. pylori* strains, J99 (ATCC 700824) and Tx30a (ATCC 51932), were used in all steps of the research. Additionally, three clinical strains of *H. pylori* (M26, M91, and M145), having dual antibiotic resistance against clarithromycin and metronidazole, were also utilized during the checkerboard assays. These strains come from the collection of microorganisms of the Wrocław Medical University in Poland and were isolated during previous studies [34]. 

All *H. pylori* strains were stored frozen at −80 °C until needed [68,69]. In order to revive them, they were sown on Columbia agars (Difco, Lublin, Poland) with 10% hemolyzed horse blood and incubated for 3 days at 37 °C under microaerophilic conditions (Genbox microaer kits, BioMerieux, Marcy I’Etoile, France) and shaking speed of 100 rpm (MaxQ 6000, Thermo Fisher, Waltham, MA, USA).

### 4.2. Assessment of the Bactericidal Activity of MYR

The determination of MICs of MYR was carried out using flat-bottom, ventilated 12-well microtiter plates (Bionovo, Legnica, Poland) [68,69]. MYR (Sigma-Aldrich, St. Louis, MO, USA) was dissolved in dimethyl sulfoxide (DMSO; Chempur, Piekary Śląskie, Poland) and a series of dilutions were made in Brain Heart Infusion broth (BHI; Oxoid, Dardilly, France) with 7% foetal calf serum (Gibco, Paisley, Scotland, UK) to obtain a gradient MYR range of 640–0.625 µg/mL and the final concentration of DMSO lower than 1% (*v/v*). Each well contained 1 mL of culture broth, the desired concentration of MYR, and 10^7^ CFU/mL of *H. pylori*. 

The MIC was taken to be the lowest concentration in which no microbial growth was observed visually after a 3-day incubation (microaerophilic, 37 °C, 100 rpm shaking). The MBC value was determined by taking 10 µL of samples from each well of the titration plate and spotting them on Columbia agars with 10% hemolyzed horse blood (3 days of microaerophilic incubation at 37 °C). Both tests determining MICs and MBCs were performed using three biological assays with three technical repetitions each. 

Independently, the assessment of the bacterial viability was performed using fluorescence microscopy (see Section “4.5. Assessment of Cell Viability”).

### 4.3. Assessment of an Inhibitory Effect of MYR on Morphological Transformation

The slowdown in morphological transformation into coccoid forms of *H. pylori* was investigated with exposure of these bacteria to various sub-MICs of MYR (40–0.625 µg/mL corresponding to 1/4×–1/256× MIC) at specific time points, i.e., after 1, 2, 3, 4, 7, and 14 days of incubation. Each well of 12-well titration plates contained 2 mL of BHI (the lack of serum served as a stress factor stimulating morphological transition) and 10^7^ CFU/mL of *H. pylori*. The well containing bacteria without MYR and well without bacteria consisted of positive and negative controls, respectively. The plates were incubated for 3 days under classical conditions (microaerophilic, 37 °C, 100 rpm shaking). 

At each time point, 50 µL of culture was used to make Gram-stained preparations [31]. From each slide, 100 bacterial cells were counted from three independent biological tests (*n* = 300). The exception was the beginning of the experiment (a 0-h time point), in which a total of 800 cells obtained from all samples were counted and presented as the mean initial number of spiral forms. The bacteria were observed under an Olympus BX50 microscope (Olympus Optical, Tokyo, Japan). 

In order to confirm the observations from light microscopy, control samples (untreated with MYR) and those exposed to 1/4× MIC of MYR, both coming from a 7-day incubation, were additionally submitted for a SEM analysis [31,68,69]. Such samples were fixed by adding a 2.5% solution of glutaraldehyde (Sigma-Aldrich) with a 1-day incubation at 4 °C. These samples were then centrifuged several times (600× *g* for 5 min) and washed in a 0.1 M cacodyl buffer (Sigma-Aldrich). After this step, the bacteria were passed through an ethanol series (50%–99.9%) and sputtered with gold. Observations were made using an Auriga 60 electron microscope (Zeiss, Oberkochen, Germany).

### 4.4. Assessment of the Interaction of MYR with Antibiotics

The potential existence of a positive interaction in the antibacterial activity of MYR with the classically used antibiotics, i.e., amoxicillin, clarithromycin, tetracycline, metronidazole, and levofloxacin (all from Sigma-Aldrich), was performed using the checkerboard assay. The research methodology was similar to that used by us previously [68,69] with minor modifications. Three selected concentrations of MYR (1/4×, 1/8×, and 1/16× MIC) and a range of antibiotic concentrations were applied during the checkerboard assays. Bacteria in the amount of 10^7^ CFU/mL were incubated in 1 mL of BHI with 7% serum (3 days of microaerophilic incubation at 37 °C, 100 rpm shaking). Since MYR alone in the concentrations used in these assays did not inhibit the growth of *H. pylori*, reading of MICs of control wells (a single compound used) was limited to antibiotics only. 

The interactions were interpreted based on the calculation of FICI, in which ≤0.5; >0.5 but ≤1, >1 was considered as synergism, additivity, and neutral interaction, respectively [68,69]. In addition, the viability of bacterial cells derived from the four wells, corresponding to the control, 1/4× MIC of MYR, MIC of antibiotics, as well as the FIC of both compounds, was determined (for analysis, see Section “4.5. Assessment of Cell Viability”).

### 4.5. Assessment of Cell Viability

The viability of *H. pylori* was examined by staining bacterial cells with the LIVE/DEAD Kit (Thermo Fisher, Waltham, MA, USA) and analyzing them by fluorescence microscopy (Olympus BX51, Olympus Optical, Tokyo, Japan) [67]. The staining procedure was performed according to the manufacturer’s recommendations. In brief, 0.1 mL of the sample was centrifuged at 10,000× *g* for 15 min, the supernatant was collected, the resulting bacterial pellet was resuspended in 1 mL of a phosphate buffer solution (PBS; Sigma-Aldrich), and then centrifuged again at the same speed. The supernatant was taken from the sample and the bacteria were suspended in 0.2 mL of a 0.85% NaCl solution with 0.6 µL of SYTO9 and propidium iodide (1:1 ratio), followed by incubation for 15 min in the dark. Then, 10 µL of samples was spotted on microscope slides and capped with coverslips. The green (viable cells) and red (degenerated cells) fluorescence were counted for 10 regions of interest from three independent bioassays (*n* = 30). The intensity of these two signals was counted separately from the same regions and then reported as the ratio of green to red fluorescence. The fluorescence analysis was performed with the ImageJ program.

### 4.6. Assessment of Biofilm Formation

Determination of the influence of selected sub-MICs of MYR (1/4×, 1/8×, and 1/16× MIC) on the formation of *H. pylori* biofilm was performed by a crystal violet method [70]. For this purpose, bacteria were incubated for 3 days under classical conditions (microaerophilic, 37 °C, 50 rpm shaking) in 1 mL of BHI broth with 2% foetal calf serum (this concentration stimulates both biofilm formation and ensures multiplication of *H. pylori*). After this period, the bacterial suspension was collected, the wells were washed twice with 1 mL PBS, dried, and stained with 1 mL of 0.1% crystal violet (Sigma-Aldrich) for 15 min. Then, the dye was removed, the wells were washed twice with PBS, dried, and flushed with 1 mL of 96% ethanol (Stanlab, Lublin, Poland) to dissolve the crystal violet adsorbed in the *H. pylori* biofilms. The 200 µL of these solutions was transferred to a 96-well microtiter plate (Bionovo, Legnica, Poland). Absorbance was measured at OD_590_ using an Asys UVM 340 microplate reader (Biochrom Ltd., Cambridge, UK). In each case, the absorbance of the negative control (wells without bacterial biofilms, being a pure culture medium) was subtracted from the absorbance of the remaining samples. The tests were performed three times in three technical repetitions.

### 4.7. Assessment of Expression of Genes Encoding Morphogenesis

The determination of the expression of genes related to the morphogenesis of MYR-exposed *H. pylori* was performed according to the procedures described by Fernandes et al. (2017) [71] with minor modifications. 

Bacteria in 10^7^ CFU/mL were grown in 12-well microtiter plates containing 1 mL BHI with 7% foetal calf serum (with 1/4× MIC of MYR or without this compound (serving as a control)) and incubated for 3 days under standard conditions (microaerophilic, 37 °C, 100 rpm shaking). After this time, the microorganisms were transferred to microcentrifuge tubes (Bionovo), centrifuged at 10,000 rpm (Eppendorf 5424R, Hamburg, Germany), the supernatant was collected, and the samples were followed to the steps provided by the manufacturer of the MINI RNA Kits (A&A Biotechnology, Gdynia, Poland). The purity and concentration of the obtained RNA was determined spectrometrically using a NanoDrop (DenoviX DS-11, Wilmington, USA) and the samples were stored at −80 °C until needed.

The TranScriba Kit (A&A Biotechnology) was applied to transcribe the obtained RNA into cDNA. The synthesis was performed in a two-step reaction with random hexamers, using incubation at 65 °C for 5 min in a T100 Thermal Cycler (Bio-Rad, CA, USA). Then, a reaction buffer, RNase inhibitor, dNTP mix, and reverse transcriptase were all added and incubated (25 °C for 5 min, then 42 °C for 60 min). The obtained cDNA was stored at −80 °C until the analysis was done. 

The qPCR reaction was performed using the RT PCR SYBR A Kit (A&A Biotechnology) and primers of the tested (*csd1, csd2, csd3, csd4, csd6*, and *amiA*) and reference genes (*ureA, gyrB*, and *glmM*), obtained from Genomed (Warsaw, Poland) (Table 2). All primer sequences were taken from Fernandes et al. (2017) [71] and used in the present study. The exception was the *csd6* gene (not present in that study), in which the primers were created by comparing the *csd6* sequences of different *H. pylori* strains, obtained from the NCBI database, and then designed using the Primer3 v.4.1 program (https://bioinfo.ut.ee/primer3/ (accessed on 10 February 2021)). The procedure was performed using a CFX Connect Real-Time PCR Detection System (Bio-Rad, CA, USA) and the listed reaction parameters, i.e., initial denaturation (98 °C for 2 min), followed by 39 cycles: denaturation (98 °C for 30 s), annealing (51–57 °C for 10 s; depending on primers in Table 2), and elongation (65 °C for 5 s). In line with the previous study, the reference gene with sufficient stability during these studies was *ureA* (the difference between the expression in the control and experimental samples was <1), which served to compare the obtained expression of the tested genes. The other two reference genes, *glmM* and *gyrB*, underwent too high changes in the expression in experimental settings compared to controls (more than 3) and were therefore not used in our research. The negative controls were wells with primers but without cDNA. Gene expression was determined by calculating 2^−∆∆Ct^ and a 2-fold change in the expression was considered as statistically significant. The tests were performed in three biological repetitions.

### 4.8. Statistical Analysis

The statistical significance of data within groups and between groups was determined by the Kruskal-Wallis test with a Bonferroni correction. For all tests, RStudio and a significance level of α = 0.05 were used.

## 5. Conclusions

In this original study, we demonstrated the potential of MYR to slow down the morphological transformation of *H. pylori* from spiral/rod-shaped forms to coccoid forms, the latter being classically involved in antibiotic tolerance and therapeutic failures. This inhibitory mechanism is most likely associated with the decreased expression of genes involved in the muropeptide monomers shortening (*csd3*, *csd6*, *csd4*, and *amiA*). Additionally, we have determined the ability of MYR to reduce the biofilm formation of *H. pylori* and obtain synergism with all five tested antibiotics (amoxicillin, clarithromycin, tetracycline, metronidazole, and levofloxacin) against this pathogen.

## Figures and Tables

**Table 1:** Antibacterial activity of myricetin against tested *Helicobacter pylori* strains.

*H. Pylori* Strains	Collection Number	Myricetin Activity [µg/mL]	
		MIC	MBC
J99	ATCC 700824	160 ± 23.1	320 ± 53.3
Tx30a	ATCC 51932	160 ± 26.7	320 ± 46.2

MIC: Minimal inhibitory concentration; MBC: Minimal bactericidal concentration. Results are presented as means ± standard deviations from three independent biological experiments with three repetitions each.

**Table 2:** Primer sequences used during the quantitative RT-PCR (RT-qPCR).

Gene	Primer Sequence (5’ to 3’)		Annealing Temperature
*csd1*	Forward	TCGCATACACAGGGGTGTTA	54 °C
	Reverse	TGCGCCTTATCCCTAATGAC	
*csd2*	Forward	CCTTTCTTTGGTGGGTTTGA	54 °C
	Reverse	GCTCTTTATTGTGGGGCAAA	
*csd3*	Forward	CGCTCATTCAAGCCCTTATC	57 °C
	Reverse	GCTAAAAGGGGGTCATTGGT	
*csd4*	Forward	TTAAACCCACCAGGCTCATC	54 °C
	Reverse	GGCTTGTGTTCTTGGGTGTT	
*csd6*	Forward	GCAGAAATTAGAGCGCTTGG	51 °C
	Reverse	GCCCTTGGTGTTCAATTCAT	
*amiA*	Forward	ATACGGTTTGCTTTGGATGC	54 °C
	Reverse	GTCCGCAAAAATTACCCTGA	
*ureA*	Forward	TTTCACGCTAACGGCTTTTT	54 °C
	Reverse	AACCGGATGATGTGATGGAT	
*glmM*	Forward	CAACCGCTTGAGAAGAAAGG	54 °C
	Reverse	CCAACCAATTAAGCCAGGAA	
*gyrB*	Forward	CGTCGCTTTGGATCATTTTT	55 °C
	Reverse	AATGGCGTGCCACTTTTAAC	

## Data Availability

Data is contained within the article.
